# Autosomal-dominant transthyretin (TTR)-related amyloidosis is not a frequent CMT2 neuropathy “in disguise”

**DOI:** 10.1186/s13023-018-0917-0

**Published:** 2018-10-04

**Authors:** Marina Grandis, Alessandro Geroldi, Rossella Gulli, Fiore Manganelli, Fabio Gotta, Merit Lamp, Paola Origone, Lucia Trevisan, Chiara Gemelli, Sabrina Fabbri, Angelo Schenone, Stefano Tozza, Lucio Santoro, Emilia Bellone, Paola Mandich

**Affiliations:** 1 Dept. of Neuroscience, Rehabilitation, Ophthalmology, Genetics and Maternal and Child Health (DINOGMI), University of Genoa, Viale Benedetto XV, Largo P. Daneo 3, 16132 Genoa, Italy; 2 Ospedale Policlinico San Martino IRCCS-Neurological Unit, Largo R. Benzi 10, 16132 Genoa, Italy; 3 Ospedale Policlinico San Martino IRCCS-Medical Genetic Unit, Largo R. Benzi 10, 16132 Genoa, Italy; 4 Department of Neurosciences, Reproductive Sciences and Odontostomatology, Federico II University of Naples, Via S. Pansini, 5, 80131 Naples, Italy

**Keywords:** TTR, CMT2, Polyneuropathy

## Abstract

Transthyretin (TTR)-related familial amyloid polyneuropathy (TTR-FAP) is a life-threatening autosomal dominant, systemic disease. First symptoms usually occur from the second to over sixth decade of life with a length-dependent axonal neuropathy with prominent involvement of the small fibers and multi-organ systemic failure.

Early diagnosis is pivotal for effective therapeutic options, but it is hampered by the heterogeneity of the clinical spectrum which can lead to misdiagnosis with other neurological condition/disorder such as axonal sensory-motor neuropathy (CMT2) as described in literature.

The aim of our study was to search for TTR mutations in a large cohort of selected undiagnosed axonal sensory-motor neuropathy patients to establish if misdiagnosis is frequent or rare in the Italian population.

No TTR pathogenic variants were found in our cohort. In conclusion, our study shows that TTR testing not should be straightforward recommended in CMT2 patients but only when “red flags” TTR’s features are present.

## Letter to the editor

Autosomal-dominant transthyretin (TTR)-related amyloidosis usually manifests in the second to over sixth decade with a length-dependent axonal neuropathy with prominent involvement of the small fibers and multi-organ systemic failure.

In Portugal and Sweden where Transthyretin Related Familial Amyloid Polyneuropathy (TTR-FAP) is endemic, disease prevalence ranges from 1 in 1.000 to 1 in 10.000 people. Beyond these endemic regions, the incidence of TTR-FAP is much lower.

More than 130 different mutations of TTR have been identified worldwide, but the first-described Val30Met mutation remains the most common with an early onset phenotype, which is typical in Portuguese population [1]. The prevalence of different mutations varies according to ethnicity and geographic region. In Italy few regions (Sicily, Puglia, Lazio, Piedmont, Tuscan-Emilian Apennines) are endemic for specific mutations.

Early diagnosis is pivotal for effective therapeutic options, but it is hampered by the heterogeneity of the clinical spectrum in not-Portuguese populations, in which the divergences from the canonical phenotype are relevant [2]. In the last years many groups described patients carrying *TTR* mutations and showing misleading clinical features [3, 4] or mimicking other neurological disorders as ALS [5, 6].

In 2011, Cappellari and colleagues reported atypical presentation of *TTR*-related familial amyloid polyneuropathy in Italian families. In their sample a relevant percentage of patients (76,4%; 13/17) received no diagnosis or a wrong diagnosis at onset, including Charcot-Marie-Tooth disease (CMT) in 2 patients.

The aim of our study was to search for *TTR* mutations in a large cohort of selected undiagnosed axonal sensory-motor neuropathy patients, classified as CMT2, to establish if CMT could occasionally mimic TTR-FAP. For this purpose, 98 consecutive patients, referred from all Italian Regions and affected with late onset axonal CMT (≥ 30 y), were enrolled at CMT Clinics of Ospedale Policlinico San Martino. Since we were looking for possible atypical *TTR* presentation, our selected cases had clinical features quite far from classic *TTR* phenotype. In particular, the mean age of onset was 46.4 years (SD: ±12,9 y; median 45 y) and the mean disease duration was 8.9 years (median: 4.5; range: some months – 53 years). Main clinical features are summarized in Table 1.

**Table 1:** Main clinical features

Total patients	98
Male	51
Female	47
Mean age	55 yr
Mean onset	46,4 ± 12,9 yr
Median onset	45 yr
Mean disease duration	8,9 ± 10,2 yr
Median disease duration	4,5 yr
Prevalent motor neuropathy	13,20%
Sensory motor neuropathy	86,80%
Hypoacusia	4%
Tremor	1%

*yr* years

All patients resulted negative for mutations in the most frequently mutated CMT2 genes (*MPZ, MNF2, GDAP1, GJB1, NEFL*). All patients underwent neurological and neurophysiological evaluation, had regular annual follow up at CMT Clinics and give informed consent to this genetic study.

No pathogenic *TTR* variants were found in this cohort.

Transthyretin-related amyloidosis is a severe multi-organ disease leading to death within 10 years after the first symptoms occur. Since several pharmacological treatments are now available [7–10], it is crucial to identify any potential case and to implement diagnostic strategies to reach an early diagnosis and plan a tailored therapy. In the early stages, a possible misdiagnosis with CIDP has been often reported and different authors recommend to consider *TTR*-related amyloidosis in cases presenting as immune-mediated neuropathies not responding to immunomodulant therapies [3].

Coincident Diabetes Mellitus and Monoclonal Gammopathy of Undetermined Significance, both frequent particularly in elder population, could also mask a *TTR*-related amyloidosis [11].

Charcot-Marie-Tooth disease is the most frequent inherited neuromuscular disorder and, in spite of a huge genetic heterogeneity, has peculiar clinical hallmarks as distal weakness and atrophy and pes cavus. The subgroup of patients presenting with a late onset axonal CMT2 could share some clinical features with *TTR* related amyloidosis, even if the course is often slowly instead of rapidly progressing.

Since Cappellari and colleagues identified a *TTR* mutation in two patients previously classified as CMT2 patients, we decided to screen a cohort of late onset CMT2 patients in which mutations in major CMT2-related genes have been excluded.

The study has some limitations. The number of CMT2 patients with a late onset, screened in this project, were referred from many Italian Regions in which *TTR* amyloidosis is not endemic and limited to 98 subjects, thus affecting the diagnostic yield.

However, although in a small sample, misdiagnosis of *TTR*-FAP in CMT2 patients seems not to be common.

In conclusion, our study shows that *TTR* testing not should be recommended in CMT2 patients but only when “red flags” TTR’s features are present, such as a rapid progressive course, carpal tunnel syndrome particularly in males, cardiologic involvement or signs suggesting autonomic nervous system impairment.

## Abbreviations

CMT: Charcot-Marie-Tooth disease
TTR: transthyretin
TTR-FAP: Transthyretin Related Familial Amyloid Polyneuropathy
